# Gradation Design of Epoxy–Asphalt Mixtures for Steel Bridge Deck Pavements Optimized for Skid Resistance in Hot and Humid Climates

**DOI:** 10.3390/polym18091088

**Published:** 2026-04-29

**Authors:** Peidong Du, Qinghua He, Zhenqiang Han, Qiang Zhang, Chuan Xiong, Yujie Zhang

**Affiliations:** 1 School of Highway, Chang’an University, Xi’an 710064, China; dawei1009@outlook.com (Q.H.); jasonhan029@126.com (Z.H.);; 2 Guangzhou Highway Co., Ltd., Guangzhou 510101, China; 3 Guangzhou Jiaotou Ruijun Real Estate Co., Ltd., Guangzhou 510288, China

**Keywords:** steel bridge deck pavement, epoxy–asphalt mixture, skid resistance, gradation design, humid and hot regions

## Abstract

To address the pronounced degradation of skid resistance in steel bridge deck pavements exposed to hot, humid, and rainy environments, this study investigates an EA-10 epoxy–asphalt mixture and proposes a gradation design method with skid resistance as the primary performance objective. An orthogonal experimental design was employed to systematically analyze different combinations of sieve passing rates, and after determining an optimum asphalt–aggregate ratio of 6.25%, the skid resistance of the mixtures under various service conditions was evaluated using macrotexture depth, dry friction coefficient, and water-film friction coefficient. The results demonstrate that the formation of skid resistance follows a mechanism in which the macroscopic framework and microscopic pores interact synergistically. The passing rate of the 4.75 mm sieve is the dominant factor governing macrotexture depth, while the 0.3 mm sieve plays a critical regulating role in texture development; meanwhile, the passing rates of the 2.36 mm and 0.6 mm sieves exert a decisive influence on both dry and water-film friction coefficients. When the passing rates of the 4.75 mm, 0.3 mm, 2.36 mm, and 0.6 mm sieves are approximately 70%, 26.5%, 58–61%, and 34%, respectively, the mixture exhibits superior overall skid-resistance performance. Based on the evaluation results of the International Friction Index (IFI), the optimized gradation shows a more stable level of skid resistance under wet and slippery conditions. These findings provide quantitative evidence and engineering guidance for the skid-resistance-oriented gradation design of epoxy–asphalt mixtures used in steel bridge deck pavements in hot and humid regions.

## 1. Introduction

In recent years, with the large-scale construction of long-span steel bridges crossing rivers and seas as well as high-grade highway bridges, the role of deck pavement within bridge structural systems has become increasingly prominent, and its service performance has emerged as a critical factor affecting overall bridge safety, durability, and riding comfort [1,2]. Steel bridge deck pavements are directly laid on steel deck plates, where the supporting layer exhibits high stiffness and limited deformation compatibility, and is highly susceptible to the coupled effects of temperature variations and traffic loading, resulting in long-term service under complex stress states and adverse environmental conditions [3,4]. Consequently, compared with conventional asphalt pavements, steel bridge deck paving materials are subject to more stringent technical requirements in terms of high-temperature stability, fatigue resistance, corrosion resistance, and skid resistance [5,6,7,8].

Epoxy–asphalt mixture is a composite material consisting of thermosetting epoxy resin as the continuous phase and asphalt as the toughening modifier. Epoxy–asphalt combines the superior bonding properties and mechanical strength of epoxy resin with the excellent flexibility and construction adaptability of asphalt materials. In recent years, it has gradually become an important material choice for steel bridge deck paving [9,10,11]. Compared with conventional base asphalt or modified asphalt materials, epoxy–asphalt forms a stable three-dimensional cross-linked network structure during curing, which endows it with pronounced advantages in strength, thermal stability, fatigue resistance, and creep resistance [12,13]. Previous studies have demonstrated that epoxy–asphalt materials exhibit high strength and durability, low curing shrinkage, and superior interfacial bonding performance, as well as excellent chemical resistance and environmental stability, enabling them to better meet the service demands of steel bridge deck pavements under high-temperature, high-humidity, and long-term repeated loading conditions [14,15,16,17].

Among the various service performance indicators of steel bridge deck pavements, skid resistance is one of the fundamental indices directly related to driving safety, and its attenuation behavior and retention capability play a decisive role in long-term pavement performance [18,19,20]. Due to the high structural stiffness, pronounced temperature-induced deformation, and frequent traffic loading of steel bridge decks, any reduction in surface skid resistance may readily trigger vehicle skidding accidents under rainy or wet conditions, posing significant safety risks. Commonly used paving materials such as SMA-10 and EA-10 in current engineering practice generally suffer from insufficient macrotexture depth and rapid degradation of surface macrotexture during long-term service, and such adverse effects on driving safety are particularly pronounced in rainy regions [21,22,23].

Existing studies have shown that, under long-term loading, the attenuation rates of skid resistance indices such as mean texture depth (MTD), mean profile depth (MPD), and British pendulum number (BPN) of epoxy–asphalt mixtures are significantly lower than those of SBS-modified asphalt mixtures, indicating superior polishing resistance and flow resistance [19]. This suggests that epoxy–asphalt mixtures possess considerable potential for maintaining skid resistance at the material level. However, skid resistance is not solely governed by binder properties, but is also closely related to the gradation structure of the mixture, resulting from the combined effects of coarse aggregate skeleton morphology, fine aggregate filling state, and surface void structure [24,25].

From a structural perspective, rational design of mixture gradation to enhance coarse aggregate interlock and optimize the load-bearing skeleton system is an effective approach to improving pavement macrotexture and skid resistance. Previous studies have indicated that variations in key sieve passing rates significantly affect the skeleton structure and surface texture characteristics of asphalt mixtures, with coarse aggregate content and certain critical sieves exerting a pronounced influence on texture depth and friction performance [26,27,28,29]. Nevertheless, most existing research has focused on a limited number of sieves or single skid resistance indices, and a systematic understanding of the functional differentiation of different particle-size sieves in skid resistance formation remains insufficient, particularly with respect to gradation optimization across the full sieve range with skid resistance as the core design objective.

In addition, skid resistance inherently exhibits multi-index and multi-condition characteristics. Macrotexture depth primarily reflects surface macrotexture features and dry friction coefficient characterizes the direct contact friction between tires and surface aggregates, whereas water-film friction coefficient is closely associated with surface void structure and drainage capacity. A single index is therefore inadequate for comprehensively evaluating skid resistance under actual service conditions. Accordingly, it is necessary to introduce a multi-index collaborative analysis approach to achieve a comprehensive assessment of skid resistance under different conditions. The International Friction Index (IFI) serves as an evaluation metric capable of comprehensively characterizing pavement texture and friction properties, providing an effective means for unified assessment of multi-indicator skid resistance. However, its application in the gradation design of epoxy–asphalt mixtures warrants further research [30,31,32,33].

Against this background, this study takes the EA-10 epoxy–asphalt mixture commonly used in steel bridge deck pavements in hot and humid regions as the research object, adopts an orthogonal experimental design to systematically configure full-range sieve passing rates between 0.075 mm and 4.75 mm, and, on the basis of determining a reasonable asphalt–aggregate ratio, evaluates skid resistance under different service conditions using macrotexture depth, dry friction coefficient, and water-film friction coefficient, while introducing the International Friction Index to analyze overall skid resistance performance. Through multi-index coordination and statistical analysis, the functional roles of different sieves in skid resistance formation are clarified, and a skid-resistance-oriented gradation design method for epoxy–asphalt mixtures suitable for steel bridge deck pavements in hot, humid, and rainy regions is proposed, providing a theoretical basis and technical reference for related engineering practice.

## 2. Materials and Methods

### 2.1. Raw Materials

#### 2.1.1. Binder Composition and Basic Performance Properties

(1) Basic properties of base asphalt:

The base asphalt used in this study was Shell 70# asphalt supplied by Guangzhou Xinyue Asphalt Co., Ltd., Guangzhou, China. In accordance with the test methods specified in *Standard Test Methods of Bitumen and Bituminous Mixtures for Highway Engineering* (JTG E20-2011) [34], fundamental properties including penetration, ductility, softening point, density, and flash point were determined. In addition, the mass change in residues after the Rolling Thin Film Oven Test (RTFOT), residual ductility, and penetration ratio were measured. The test results are summarized in Table 1.

(2) Basic properties of epoxy resin and curing agent:

The epoxy binder adopted in this study was the KD-BEP epoxy resin produced by Kindai Chemical Co., Ltd., Tokyo, Japan. Among these, the epoxy resin base is bisphenol A epoxy resins (DGEBA), and the curing agent is a modified amine curing agent. Based on the product technical specifications and standard testing procedures, the primary performance properties of the epoxy resin and curing agent were evaluated, and the results are presented in Table 2 and Table 3.

#### 2.1.2. Basic Properties and Mechanical Volume Parameters of Aggregates

The coarse aggregates were sourced from the Shiniuling Quarry, while the fine aggregates consisted of basalt-manufactured sand supplied by the Guangdong Changda Zhongshan Aggregate Plant. Mineral filler was provided by Luoding Xinda Aggregate Plant. The basic properties of the aggregates were tested in accordance with JTG E42-2005 [37], with the results reported in Table 4, Table 5 and Table 6.

It is noteworthy that, in addition to the conventional volume and mechanical parameters listed in the table, the fine aggregate’s high sand equivalent (88%) and low methylene blue value (0.8 g/kg) indirectly indicate its fine powder fraction possesses favorable particle size distribution and surface cleanliness. This provides a microscopic foundation for achieving high bulk density in the mixture. The hydrophobic properties of the mineral powder (hydrophilic coefficient of 0.81) facilitate the formation of a stable interfacial transition zone on the aggregate surface, enhancing the bond between epoxy–asphalt and aggregates. This is crucial for improving the workability of the mixture and its subsequent resistance to water damage in humid and hot environments.

### 2.2. Experimental Methods

#### 2.2.1. Specimen Preparation

(1) Preparation of Marshall specimens:

The epoxy resin and curing agent were conditioned at 60 °C for 3 h to enhance fluidity, after which they were thoroughly mixed at a mass ratio of 56:44. The blended epoxy resin was then combined with the base asphalt at a ratio of 1:1 to form the epoxy–asphalt binder.

According to the designed gradation, the asphalt binder heated to 165 °C was mixed uniformly with aggregates heated to 180 °C and then placed into standard Marshall molds. Compaction was performed using a hammer of specified mass and drop height, applying 50 blows to each side of the specimen to ensure uniform densification. After compaction, specimens were cured at 120 °C for 4 h, followed by curing at 60 °C for 4 days before demolding. The prepared specimens were required to meet specified requirements in terms of dimensions, mass, and surface integrity.

(2) Preparation of slab specimens:

Asphalt mixtures were mixed at 180 °C according to the designed gradation and rapidly placed into molds preheated to the forming temperature for paving. The specimens were then compacted using a wheel-rolling compactor with 12 reciprocating passes to achieve the target density. After molding, the slabs were cured at 120 °C for 4 h and subsequently at 60 °C for 4 days before demolding, and were used for friction coefficient and macrotexture depth measurements.

#### 2.2.2. Testing Methods

(1) Density measurement of compacted asphalt mixtures:

The apparent density of the specimens was determined using the saturated surface-dry method. First, the mass of the dry specimen in air was measured and recorded as mₐ. The specimen was then immersed in flowing water at 25 ± 0.5 °C for 3–5 min to remove entrapped air, and the submerged mass was measured as m_w_. After removal, the surface water film was quickly wiped off with a damp cloth without extracting pore water, and the mass was recorded as m_f_. The apparent density of the specimen was calculated using the following equation:

(1) $$\gamma_{f}=\frac{m_{a}}{m_{f}-m_{w}}$$

(2) $$\mathrm{VV}=(1-\frac{\gamma_{f}}{\gamma_{t}})\times 100$$

(3) $$\mathrm{VMA}=(1-\frac{\gamma_{f}}{\gamma_{\mathrm{sb}}}\cdot \frac{P_{s}}{100})\times 100$$

(4) $$\mathrm{VFA}=\frac{\mathrm{VMA}-\mathrm{VV}}{\mathrm{VMA}}\times 100$$

where $\gamma_{f}$ denotes the bulk density of the specimen (g/cm^3^); m_a_ is the dried mass of the specimen (the mass measured in air at the dry state) (g); m_f_ is the surface dry mass of the specimen (the mass measured in air after removing it from water and wiping off the surface water film) (g); m_w_ is the specimen’s mass in water (the mass measured when submerged in water) (g); VV represents the air void content of the asphalt mixture (%); $\gamma_{t}$ is the maximum theoretical density of the asphalt mixture (g/cm^3^); VMA denotes the voids in mineral aggregate (%); $\gamma_{\mathrm{sb}}$ is the apparent density of the aggregate (g/cm^3^); P_s_ is the mass percentage of mineral aggregate in the asphalt mixture (%); and VFA represents the percentage of voids in the mineral aggregate filled with asphalt (%).

(2) Marshall stability and flow test:

The deformation resistance of the asphalt mixtures was evaluated using the Marshall stability and flow test. Standard specimens, prepared and cured to room temperature, were immersed in a water bath at 60 ± 1 °C for 30~40 min to ensure uniform internal temperature. The specimens were then removed and immediately placed into the testing fixture, where an axial compressive load was applied at a loading rate of 50 mm/min until failure occurred. The maximum load sustained during loading was recorded as the Marshall stability, and the corresponding deformation was measured as the flow value. During testing, proper centering of the specimen was ensured to prevent errors caused by eccentric loading.

(3) Friction coefficient measurement:

At an ambient temperature of 20 °C, the friction coefficient was measured using a British pendulum tester. Flat, clean, and dry surface areas were selected as test locations. After adjusting the apparatus to allow the pendulum to freely swing past the horizontal position, the pointer reading after passing over the test surface was recorded. Subsequently, a quantified amount of purified water was sprayed onto the specimen surface to form a water film with a thickness of 0.03 mm, and the corresponding friction coefficient was measured. Each test location was measured no fewer than five times, and the arithmetic mean was taken as the final value, reported as an integer.

(4) Macrotexture depth measurement:

The pavement macrotexture depth was determined using the sand patch method. A volume of 25 cm^3^ of standard sand was uniformly spread onto a clean and dry test surface. A circular disk was then used to distribute the sand evenly in a circular motion until a well-defined circular patch was formed, and the maximum and minimum diameters were measured and averaged. The macrotexture depth was calculated by dividing the sand volume by the area of the sand circle. During testing, the effects of wind and moisture were carefully avoided to ensure accurate and reliable measurements.

## 3. Results and Discussion

### 3.1. Design of the Orthogonal Experimental Scheme

To systematically investigate the effects of key sieve passing rates on the skid resistance of epoxy–asphalt mixtures and to efficiently optimize mixture gradation, this section takes the EA-10 epoxy–asphalt mixture commonly used in steel bridge deck pavements as the research object and designs the gradation composition using an orthogonal experimental method. This approach enables the primary and secondary effects as well as the influence patterns of multiple factors to be scientifically analyzed with a minimum number of experimental runs.

Seven sieve sizes—0.075, 0.15, 0.3, 0.6, 1.18, 2.36, and 4.75 mm—that play key controlling roles in the EA-10 gradation were selected as experimental factors. Among them, six sieves (0.075, 0.15, 0.3, 0.6, 1.18, and 4.75 mm) primarily govern the composition of fine aggregates and mineral filler, and three levels were selected for each factor within the corresponding design gradation range (based on upward and downward deviations from the median values specified in JTG/T 3364-02-2019) [38], representing lower-limit, median, and upper-limit passing rates. The 2.36 mm sieve was regarded as the critical boundary distinguishing coarse and fine aggregates, and six levels were specifically assigned to this sieve to enable refined analysis. The detailed factor-level design is presented in Table 7.

Each gradation scheme was used to prepare epoxy–asphalt mixture slab specimens, and the British pendulum number (BPN) and mean texture depth (MTD) were measured as response indices for skid resistance evaluation. Subsequently, a combination of range analysis and analysis of variance (ANOVA) was employed to process the experimental data. Range analysis was used to intuitively determine the relative importance of each factor on BPN and MTD and to preliminarily identify the optimal factor levels, while ANOVA was conducted at a significance level of α = 0.05 to quantitatively assess the statistical significance of factor effects, thereby providing a rigorous basis for gradation optimization.

### 3.2. Determination of the Optimum Asphalt–Aggregate Ratio

**Figure 1:**
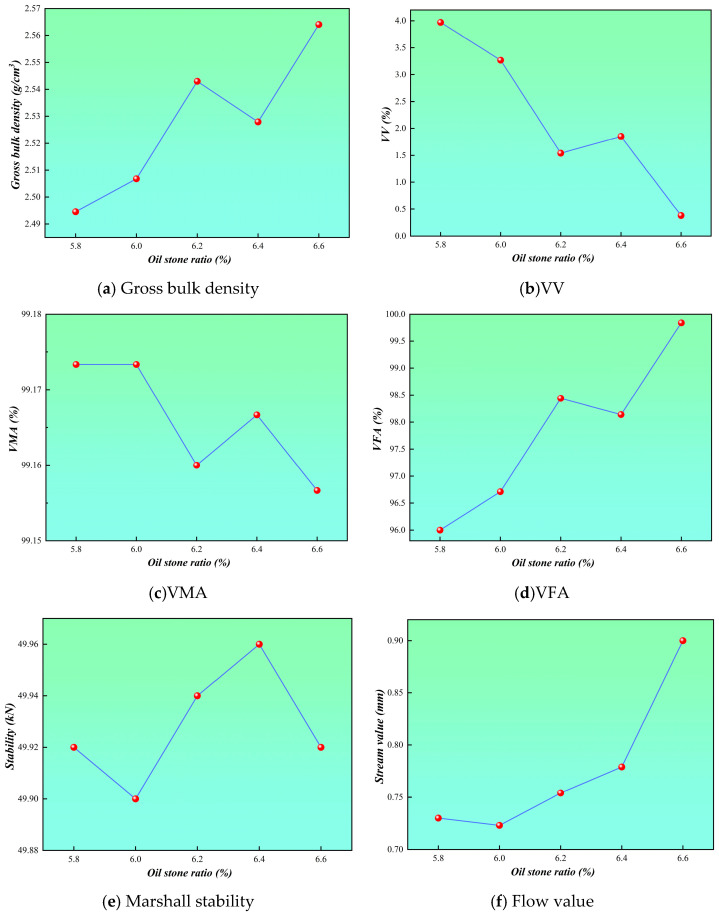
Determine the optimal asphalt dosage.

Based on performance testing of Marshall specimens prepared with different asphalt–aggregate ratios (5.8%, 6.0%, 6.2%, 6.4%, and 6.6%), the relationships between asphalt–aggregate ratio and bulk density, air voids (VV), voids in mineral aggregate (VMA), voids filled with asphalt (VFA), Marshall stability, and flow value were plotted and analyzed, as shown in Figure 1. The results indicate that bulk density initially increases and then decreases with increasing asphalt–aggregate ratio, reaching a maximum within the range of 6.2–6.4%. The air void content decreases with increasing asphalt content while remaining within the specification limits. VMA exhibits a relatively stable trend and satisfies design requirements, whereas VFA increases with asphalt content, indicating effective asphalt filling. Marshall stability reaches its peak at approximately 6.2%, while the flow value increases with asphalt–aggregate ratio and remains within the allowable range specified by standards. By comprehensively considering the variation trends of all performance indicators and relevant specification requirements, the optimum asphalt–aggregate ratios corresponding to the upper-limit, median, and lower-limit gradations of the selected factor levels were determined to be 6.20%, 6.30%, and 6.25%, respectively. The optimal oil-to-aggregate ratio for the three gradations falls within a narrow range of 6.20% to 6.30%, with a mere 0.10% variation. This indicates that within this gradation range, the asphalt mixture exhibits low sensitivity to the oil-to-aggregate ratio, demonstrating a broad “optimal oil-to-aggregate ratio plateau zone.” To balance the comprehensive road performance of the three gradations and ensure the feasibility of subsequent rutting tests requiring a single oil-to-aggregate ratio for comparative analysis, this study selected the median value of 6.25% as the final optimal oil-to-aggregate ratio. All 20 gradations were prepared using this asphalt–aggregate ratio to produce rutting specimens for skid resistance testing.

It should be noted that this study uniformly employed an oil-to-aggregate ratio of 6.25% during the orthogonal experimental design phase. This was done to eliminate the confounding effect of asphalt content, thereby focusing on the main effects of gradation factors on skid resistance and providing a comparable performance benchmark for all test gradations. However, there is an inherent interdependence between the optimal oil-to-stone ratio and the aggregate gradation. Therefore, the optimized gradation identified in this paper under a fixed oil-to-stone ratio should be regarded as a relatively optimal solution under specific conditions.

### 3.3. Influence of Gradation on Macrotexture Depth Based on Orthogonal Experiments

Macrotexture depth is a key indicator characterizing the richness of pavement surface macrotexture and directly reflects the average depth of the uneven surface structure formed by aggregate particles [39]. As one of the core factors governing pavement skid resistance, macrotexture depth primarily functions by providing rapid drainage pathways for water accumulated at the tire–pavement interface, thereby effectively reducing the occurrence of water-film-induced skidding during high-speed vehicle operation and enhancing driving safety [40,41].

The macrotexture depth was calculated using the following equation:

(5) $$\mathrm{TD}=\frac{1000V}{\pi D^{2}/4}$$

where TD denotes the surface macrotexture depth of the asphalt mixture (mm), V is the volume of sand (25 cm^3^), and D is the average diameter of the flattened sand patch (mm).

The measured macrotexture depths corresponding to each orthogonally designed gradation scheme are listed in the following Table 8.

#### 3.3.1. Range Analysis Results and Discussion on Macrotexture Depth

Range analysis was conducted by calculating the mean macrotexture depth (*Kᵢ*) at each factor level and the corresponding range (*R*, defined as *K*_max_ − *K*_min_). The magnitude of *R* directly reflects the extent to which variations in factor levels influence the response index; a larger *R* value indicates a more pronounced effect of the factor on macrotexture depth. Based on the gradation schemes and corresponding macrotexture depth data presented in Table 8, the mean values and ranges of each factor were calculated, as summarized in Table 9.

The range analysis results reveal substantial differences among the R values of the investigated factors, indicating that the effects of different sieve passing rates on macrotexture depth are not equivalent. The relative influence of the factors on macrotexture depth can be ranked as follows: Factor A (4.75 mm) > Factor E (0.3 mm) > Factor B (2.36 mm) > Factor G (0.075 mm) > Factor C (1.18 mm) > Factor D (0.6 mm) = Factor F (0.15 mm). Among all factors, the 4.75 mm sieve passing rate exhibits the largest range value (R = 0.041 mm), which is markedly higher than those of the other sieves, indicating that variations in coarse aggregate skeleton proportion play a dominant role in governing pavement surface macrotexture characteristics [42]. As the 4.75 mm passing rate increases from 70% to 75% and 80%, the mean MTD decreases sequentially from 0.725 mm to 0.691 mm and 0.684 mm, demonstrating a clear decreasing trend with increasing passing rate. This indicates that a relatively higher proportion of coarse aggregates is conducive to the formation of a well-developed interlocking skeleton structure and pronounced macrotexture [43].

The passing rate of the 0.3 mm sieve also shows a pronounced influence on macrotexture depth, with a range value of 0.029 mm. The highest mean MTD (0.714 mm) is observed at Level 2 (26.5%), whereas deviations from this level result in a reduction in macrotexture depth. This indicates the existence of an optimal content range for this fine aggregate fraction, where moderate filling helps maintain surface texture continuity, while either excessive or insufficient fine aggregate content is detrimental to macrotexture development [44].

The remaining sieve factors exhibit relatively limited effects on macrotexture depth, with all range values below 0.020 mm; among them, the 0.6 mm and 0.15 mm sieves show the smallest ranges (both 0.009 mm). These fine aggregate and filler fractions mainly function in regulating mixture densification and contribute less directly to the formation of surface macro-relief features.

Based on the range analysis results, optimization of macrotexture depth should primarily focus on the 4.75 mm and 0.3 mm sieve passing rates. It is recommended that the 4.75 mm passing rate be controlled at approximately 70%, while the 0.3 mm passing rate be maintained near 26.5%, so as to achieve an optimal balance between coarse aggregate interlocking and fine aggregate filling effects. These findings provide a preliminary basis for subsequent significance testing through analysis of variance.

#### 3.3.2. Analysis of Variance and Significance Testing for Macrotexture Depth

To further quantitatively determine the influence of sieve passing rates on macrotexture depth (MTD), analysis of variance (ANOVA) was conducted on the experimental results following the range analysis. The ANOVA results are presented in Table 10.

The ANOVA results indicate that the effects of different factors on macrotexture depth vary significantly. As shown in Table 10, the F-test result for the 4.75 mm sieve passing rate (Factor A) reaches statistical significance, with a *p*-value below 0.05, indicating that this factor has a statistically significant effect on macrotexture depth at α = 0.05. The 0.3 mm sieve passing rate (Factor E) also passes the significance test, confirming its significant contribution to macrotexture depth. In contrast, the sieve factors of 2.36 mm, 1.18 mm, 0.6 mm, 0.15 mm, and 0.075 mm do not reach the required level of statistical significance, indicating that within the investigated factor ranges, their effects on macrotexture depth are insufficient to exceed the variability attributable to experimental error.

These findings are consistent with the conclusions drawn from the range analysis, namely that the 4.75 mm sieve acts as the dominant controlling factor and the 0.3 mm sieve serves as an important influencing factor, while the effects of other sieves are relatively limited. This further demonstrates that macrotexture depth is primarily governed by the combined effects of coarse aggregate skeleton structure and critical fine aggregate filling, whereas intermediate and finer sieve sizes contribute less directly to macrotexture formation [42,45].

From a mechanistic perspective, the passing rate of the 4.75 mm sieve directly determines the proportion and interlocking state of coarse aggregates in the mixture, and its variation essentially reflects a transition of the macroscopic skeleton structure from an “interlocked–void” type to a “filled–dense” type [45]. When the 4.75 mm passing rate is relatively low, coarse aggregates are able to form a stable interlocking skeleton structure, resulting in pronounced surface undulations and well-preserved macrotexture features, thereby yielding a larger macrotexture depth. As the passing rate increases, the relative proportion of coarse aggregates decreases, the skeleton system is weakened, surface relief diminishes accordingly, and macrotexture depth exhibits a decreasing trend. Because this process involves an overall reconstruction of the skeleton system, its influence on macrotexture depth exhibits pronounced structural characteristics and therefore manifests as a significant effect in the ANOVA results.

The statistical significance of the 0.3 mm sieve passing rate reflects the “critical filling effect” of fine aggregates in the formation of pavement macrotexture. Particles retained on the 0.3 mm sieve fall within the most sensitive size range for filling skeleton voids in the fine aggregate system, and variations in their content significantly affect the degree of void filling and the stability of surface texture. When the passing rate of this sieve falls within a reasonable range, fine aggregates can enhance structural stability without disrupting the coarse aggregate interlocking skeleton, thereby maintaining continuous macrotexture. However, when the content becomes excessive, the filling effect is markedly intensified, skeleton voids are substantially filled, surface relief is weakened, and macrotexture depth consequently decreases. As this process directly affects the stability of the skeleton–void system, its statistical effect likewise appears as significant in the ANOVA results [46].

In contrast, intermediate sieve sizes such as 2.36 mm, 1.18 mm, and 0.6 mm, as well as very fine sieves such as 0.15 mm and 0.075 mm, do not pass the significance test within the investigated factor ranges. This indicates that these factors mainly function in local gradation adjustment or micro-scale filling, with relatively limited influence on macrotexture formation. In particular, very fine aggregate sieves primarily affect the densification of the mastic–fine aggregate system and surface smoothness, and their influence on macrotexture depth often manifests as a weakening tendency. However, due to their limited adjustment magnitude and the fact that macrotexture is predominantly governed by the coarse aggregate skeleton, their effects are difficult to detect as statistically significant.

Overall, the ANOVA results indicate that the 4.75 mm sieve passing rate is the primary controlling factor for macrotexture depth, the 0.3 mm sieve passing rate is an important influencing factor, and the remaining sieves contribute relatively little within the investigated experimental range. Taken together, the results of range analysis and ANOVA demonstrate that gradation structure has a significant effect on pavement surface macrotexture, with the coarse aggregate skeleton acting as the dominant controlling factor and key fine aggregate sieve passing rates serving an important regulating role. In engineering applications and gradation optimization, priority should be given to controlling the 4.75 mm sieve passing rate to ensure the stability of the coarse aggregate skeleton, while synergistically adjusting macrotexture through appropriate regulation of the 0.3 mm sieve passing rate to avoid texture blunting caused by excessive fine aggregate filling. The remaining sieves may be coordinated under the premise of satisfying workability and structural uniformity requirements, thereby balancing macrotexture formation and overall gradation stability and providing a solid foundation for subsequent multi-index collaborative optimization.

### 3.4. Influence of Gradation on Dry Friction Coefficient Based on Orthogonal Experiments

Compared with macrotexture depth, the friction coefficient represents the ratio of tangential to normal forces at the tire–pavement contact interface and constitutes an integrated response combining adhesion governed by surface macrotexture, hysteresis/damping associated with macrotexture, and intrinsic material properties. As such, it directly determines vehicle braking efficiency, driving stability, and directional controllability [47]. The measured dry friction coefficients for the investigated gradation schemes are presented in Table 11.

#### 3.4.1. Range Analysis Results and Discussion on Dry Friction Coefficient

Based on the dry friction coefficient results shown in Table 11, range analysis was conducted on the 20 orthogonal experimental datasets to clarify the effects of different sieve passing rates on the skid resistance of epoxy–asphalt mixtures under dry conditions. The calculated ranges for each factor are summarized in Table 12.

The relative influence of the factors on the dry friction coefficient is ranked as follows: Factor B (2.36 mm) > Factor D (0.6 mm) > Factor E (0.3 mm) > Factor G (0.075 mm) > Factor C (1.18 mm) = Factor F (0.15 mm) > Factor A (4.75 mm).

The 2.36 mm sieve passing rate exhibits a remarkably high range value of 11.7, far exceeding those of the other factors, indicating that this boundary sieve plays a decisive role in governing dry friction performance. At Level 3 (58%), the mean dry friction coefficient reaches 78.5, which is significantly higher than those at the remaining levels; however, when the passing rate increases to 64% or above, the mean value decreases to the range of 66.83–71.88. This behavior can be attributed to the regulatory role of the 2.36 mm particle size in controlling the proportion of course and fine aggregates. A moderate passing rate facilitates the formation of a stable interlocking structure of coarse aggregates in the surface layer, with sufficient exposure of aggregate angularity, thereby enhancing mechanical interlock between the tire and pavement. In contrast, excessively high passing rates increase the fine aggregate content, and the intensified filling effect suppresses surface roughness features, leading to a reduction in friction performance [48,49].

The 0.6 mm sieve passing rate shows the second-largest range value (9.85). At Level 1 (30%), the mean dry friction coefficient reaches 77.42, and then decreases sequentially to 70.79 at 34% and 67.57 at 38% as the passing rate increases. The 0.6 mm particle size lies within the transition zone between coarse and fine aggregates, and variations in its content directly affect the compactness of the surface structure and the continuity of surface texture. At lower passing rates, skeleton voids are adequately preserved, allowing more effective transmission of friction forces during tire contact; when the content becomes excessive, cooperative filling with fine aggregates is intensified, suppressing aggregate angularity exposure and resulting in reduced friction performance.

#### 3.4.2. Analysis of Variance and Significance Testing for Dry Friction Coefficient

To further quantitatively assess the influence of sieve passing rates on the dry friction coefficient, analysis of variance (ANOVA) was conducted on the experimental results following the range analysis. The ANOVA results are presented in Table 13.

The results indicate significant differences in the effects of different sieve passing rates on the dry friction coefficient. Specifically, the F-test results for the 2.36 mm sieve (Factor B) and the 0.6 mm sieve (Factor D) pass the significance test, demonstrating statistically significant effects at the α = 0.05 level. The remaining sieve factors do not reach statistical significance, indicating that their contributions to dry friction performance are relatively limited within the investigated factor ranges.

The dry friction coefficient primarily originates from direct contact and shear interaction between the tire and pavement aggregates. When the 2.36 mm passing rate falls within an appropriate range, coarse aggregates are able to form a relatively stable interlocking structure in the surface layer, with sufficient exposure of aggregate angularity and an increased number of effective tire–pavement contact points, thereby promoting higher dry friction coefficients. Because this sieve passing rate directly governs the morphology of the surface skeleton, its effect on dry friction performance manifests as statistically significant in the ANOVA results.

The statistical significance of the 0.6 mm sieve passing rate reflects the critical regulating role of intermediate-sized aggregates in dry friction formation. Particles retained on the 0.6 mm sieve are located in the transition zone between coarse and fine aggregates, and variations in their content markedly affect surface structural compactness and texture continuity. An appropriate amount of 0.6 mm aggregate helps stabilize the surface structure without disrupting the coarse aggregate interlocking skeleton, enabling more continuous friction force transmission during tire contact and thereby enhancing the dry friction coefficient. Conversely, excessive content intensifies the filling effect in combination with fine aggregates, suppresses surface roughness features, and leads to a decline in dry friction performance. Owing to this pronounced “regulation–amplification” mechanism, its effect also appears as statistically significant in the ANOVA.

In contrast, the 4.75 mm and 1.18 mm sieves, as well as fine aggregate sieves smaller than 0.3 mm, do not pass the significance test, indicating that these factors mainly affect macrotexture morphology or the filling state of the mastic–fine aggregate system, and exert a relatively limited controlling effect on direct tire–aggregate contact friction under dry conditions.

#### 3.4.3. Integrated Analysis of Dry Friction Coefficient and Gradation Optimization Direction

The combined results of range analysis and ANOVA indicate that, unlike macrotexture depth which is dominated by macroscopic skeleton control, the dry friction coefficient primarily reflects direct contact friction between the tire and pavement aggregates, and its formation mechanism is jointly governed by aggregate angularity exposure, the number of effective contact points, and surface structural stability.

The analysis results demonstrate that the dry friction coefficient is jointly governed by two key sieves, namely 2.36 mm and 0.6 mm. The passing rate of the 2.36 mm sieve acts as the primary controlling factor, while the 0.6 mm sieve plays an important regulating role. Together, they determine the morphology of the surface skeleton structure and the continuity of contact friction.

From the perspective of gradation optimization, improvement of the dry friction coefficient should focus on coordinated control of the two key sieves, 2.36 mm and 0.6 mm. The 2.36 mm passing rate is recommended to be controlled at approximately 58% to ensure adequate skeleton interlock and angularity exposure, while the 0.6 mm passing rate should be maintained at around 30% to avoid excessive filling. The remaining sieves, including the 4.75 mm sieve, may be configured in a coordinated manner provided that basic skeleton requirements and structural uniformity are satisfied, thereby balancing workability and multi-objective performance optimization.

### 3.5. Influence of Gradation on Water-Film Friction Coefficient Based on Orthogonal Experiments

This study further investigates the friction coefficient of epoxy–asphalt mixtures under surface water-film conditions. This condition simulates rainy or wet pavement environments and more realistically reflects skid resistance performance under scenarios with heightened safety risks. The water-film friction coefficient primarily characterizes pavement drainage capability under rainfall conditions and tire–pavement contact characteristics, and is particularly sensitive to gradation structure. Therefore, targeted analysis of this index is of substantial engineering significance.

The measured friction coefficients of each gradation under a 0.03 mm surface water film are summarized in Table 14.

#### 3.5.1. Range Analysis Results and Discussion on Water-Film Friction Coefficient

Based on the orthogonal experimental results, range analysis was conducted on the friction coefficients of epoxy–asphalt mixtures under a 0.03 mm surface water film, with the results presented in Table 15.

The analysis indicates that the relative influence of the factors on the water-film friction coefficient follows the order: Factor B (2.36 mm) > Factor D (0.6 mm) > Factor E (0.3 mm) > Factor A (4.75 mm) > Factor G (0.075 mm) > Factor F (0.15 mm) > Factor C (1.18 mm). This ranking is highly consistent with that obtained for the dry friction coefficient, indicating that the dominant controlling factors for skid resistance remain the same under both dry and wet conditions.

The 2.36 mm sieve passing rate exhibits the largest range value (12.3), identifying it as the most critical factor affecting the water-film friction coefficient. At Level 3 (58%), the mean value reaches 74.33, which is significantly higher than those at other levels; however, when the passing rate increases to 61% or above, the mean value decreases to the range of 62.17–68.25. The 0.6 mm sieve passing rate ranks second, with a range value of 10.7. The highest mean value (73.17) occurs at Level 2 (34%), exceeding those at Level 1 (66.83) and Level 3 (62.50). Unlike the monotonic decreasing trend observed under dry conditions, the 0.6 mm passing rate under water-film conditions exhibits an initial increase followed by a decrease. Among the remaining factors, the range values for the 0.3 mm, 0.075 mm, 0.15 mm, and 1.18 mm sieves are 4.00, 1.08, 1.29, and 1.01, respectively, indicating relatively limited contributions to water-film friction performance.

#### 3.5.2. Analysis of Variance and Significance Testing for Water-Film Friction Coefficient

To statistically examine the significance of the effects of different sieve passing rates on the water-film friction coefficient and further verify the conclusions drawn from the range analysis, analysis of variance (ANOVA) was conducted on the experimental results. The ANOVA results are presented in Table 16.

The ANOVA results indicate that the effects of the 2.36 mm and 0.6 mm sieve passing rates on the water-film friction coefficient reach statistical significance, with corresponding *p*-values of 0.035 and 0.013, respectively, confirming their dominant roles from a statistical perspective.

From a structural mechanism perspective, variations in the 2.36 mm sieve passing rate directly affect the surface skeleton structure and pore system of the mixture. When this passing rate lies within an appropriate range, coarse aggregates form a relatively stable interlocking skeleton, and the skeleton voids maintain sufficient connectivity to provide drainage pathways for water-film removal, thereby mitigating the isolating effect of the water film at the tire–pavement interface. Conversely, excessively high passing rates lead to filling of skeleton voids by fine aggregates, obstruction of drainage pathways, increased water retention, and deterioration of the water-film friction coefficient.

The statistical significance of the 0.6 mm sieve passing rate indicates that intermediate-sized aggregates play an important regulating role in skid resistance under wet conditions. Variations in the content of this particle size affect pore size distribution and near-surface structural characteristics. An appropriate amount of 0.6 mm aggregate helps maintain continuity and stability of the pore structure, thereby enhancing drainage efficiency; however, excessive content tends to intensify the filling effect in combination with fine aggregates, weakening pore connectivity and adversely affecting water-film removal.

In contrast, the passing rates of the 4.75 mm and 1.18 mm sieves, as well as the 0.15 mm and 0.075 mm sieves, do not pass the significance test, indicating that their effects on the water-film friction coefficient are not statistically significant within the investigated factor ranges. Their influence is mainly reflected in indirect regulation of macroscopic structure or micro-scale filling states, with limited direct contribution to water-film disruption under wet conditions.

#### 3.5.3. Integrated Analysis of Water-Film Friction Coefficient and Gradation Optimization Direction

The combined results of range analysis and ANOVA indicate that the formation mechanism of water-film friction performance differs from that of dry friction, which relies primarily on aggregate contact characteristics, and instead places greater emphasis on the coordinated control of surface pore connectivity and drainage capacity.

The influence of gradation structure on the water-film friction coefficient is mainly manifested along two dimensions: intermediate particle sizes and the skeleton boundary sieve. Among these, the 0.6 mm sieve passing rate exhibits the strongest statistical response and serves as the key controlling factor for maintaining drainage pathway continuity and surface drainage capacity. The 2.36 mm sieve passing rate acts as the dominant factor in skeleton formation and further affects the openness of the surface pore system by regulating the coarse-to-fine aggregate ratio and interlocking structure. Although the 0.3 mm sieve does not reach statistical significance, it consistently shows a secondary influence in multiple analyses, indicating that fine aggregate filling still plays a certain regulating role in water-film removal.

In gradation design, priority should be given to the 0.6 mm and 2.36 mm sieves as the core control parameters. The 0.6 mm passing rate is recommended to be maintained within a range that effectively regulates drainage pathway functionality, with an optimal value around 34%, while the 2.36 mm passing rate should be kept near 58% to enhance connectivity of the surface drainage structure. For the 0.3 mm sieve and finer particles, passing rates may be controlled under the premise of satisfying workability and compaction requirements, so as to prevent excessive fine content from increasing the risk of water-film retention and to achieve coordinated optimization of drainage efficiency and structural stability.

### 3.6. Integrated Analysis of Three Performance Indicators and Final Gradation Optimization Recommendations

Comprehensive analysis of the orthogonal experimental results for macrotexture depth, dry friction coefficient, and water-film friction coefficient reveals that although the dominant controlling factors and significance test outcomes differ among the three indicators, clear synergistic characteristics are observed.

Macrotexture depth is mainly controlled by the 4.75 mm and 0.3 mm sieves. The 4.75 mm passing rate directly affects the amplitude of pavement surface macro-relief, while the 0.3 mm passing rate indirectly influences texture continuity by regulating the degree of fine aggregate filling.

The dry and water-film friction coefficients exhibit highly consistent dominant control patterns, wherein variations in the 2.36 mm passing rate directly regulate the interlocking state of the surface skeleton and the degree of aggregate angularity exposure, thereby affecting tire–pavement contact friction characteristics. The role of the 0.6 mm sieve differs between dry and wet conditions. Under dry conditions, a lower passing rate favors preservation of skeleton voids and enhances continuity of friction force transmission, whereas under wet conditions, an intermediate passing rate (34%) optimizes pore size distribution and achieves a balance between drainage efficiency and structural stability. Accordingly, gradation design should be adjusted in a targeted manner depending on the dominant service condition.

Based on these findings, a hierarchical control and synergistic optimization strategy for gradation design is proposed. The 2.36 mm sieve is selected as the core control parameter, with its passing rate maintained within the range of 58–61% to balance skeleton stability and pore openness. The 0.6 mm sieve should be configured in a differentiated manner according to service conditions, with approximately 30% recommended for dry-dominated scenarios and about 34% for wet-dominated scenarios. The 4.75 mm and 0.3 mm sieves should be jointly controlled to achieve macrotexture objectives, with optimal values near 70% and 26.5%, respectively, to ensure a solid macrotexture foundation. The remaining sieves may be flexibly adjusted based on material characteristics and engineering experience, provided that workability and volumetric performance requirements are satisfied.

This strategy overcomes the limitations of traditional gradation design approaches that rely primarily on single indicators or empirical judgment by quantitatively defining the functional roles and interactions of different sieves, thereby providing a systematic framework for refined skid-resistance control of steel bridge deck pavements in hot and humid regions. In practical applications, moderate adjustments to key sieve passing rates may be made in consideration of local climate conditions, traffic composition, and maintenance strategies, so as to achieve a balanced optimization of performance and cost.

To further illustrate the advantages of this method, a qualitative comparison with traditional grading design methods is presented below, focusing on three aspects: design philosophy, understanding of sieve aperture functions, and optimization logic.

In terms of design philosophy, both the traditional Marshall method and the Superpave method focus primarily on volume parameters and mechanical properties, treating skid resistance merely as a post-construction verification criterion, thereby lacking targeted consideration during the design phase. Although the SAC method focuses on texture depth, its assumption that higher macrotexture depth correlates with better skid resistance does not adequately explain the experimental findings in this study, where the MTD was highest for Grade 4 aggregate, while the IFI exhibited the lowest value. This method establishes skid resistance as a direct objective of gradation design and uses the simultaneous optimization of multiple skid resistance indicators under various operating conditions as its guiding principle, thereby addressing the shortcomings of traditional methods at the design level.

With regard to understanding the functions of sieve apertures, traditional methods do not clearly define the specific roles of different particle sizes; they typically only distinguish between coarse and fine aggregates, and lack a quantitative understanding of the independent role of fine aggregates in providing skid resistance on wet and slippery surfaces. Through quantitative analysis, this method identifies the distinct roles various sieve apertures play in the formation of skid resistance: the 4.75 mm sieve size dominates the macroscopic framework; the 2.36 mm sieve size determines the degree of aggregate edge exposure and frictional contact; while the 0.6 mm and 0.3 mm sieve sizes work in concert to construct a connected microscopic drainage network, elevating the function of fine aggregates from mere filling to that of microscopic drainage construction.

In terms of optimization logic, traditional methods often rely on empirical debugging or single-parameter adjustments, and are unable to account for the constraints and balances among multiple metrics. This method quantitatively determines the priority ranking and optimal configuration ranges for each key aperture, facilitating a shift from experience-based debugging to a mechanism-driven, multi-objective optimization approach.

In summary, this method improves upon traditional approaches in terms of design philosophy, functional understanding, and optimization logic. The proposed mechanism for the coordinated control of macroscopic framework and microscopic porosity provides a general methodological framework for the design of skid-resistant gradations in asphalt mixtures.

### 3.7. International Friction Index (IFI)

Although macrotexture depth and friction coefficient exhibit a certain degree of correlation, their discrete representations cannot jointly and in a standardized manner reflect the overall skid resistance of a pavement. Therefore, this study introduces the International Friction Index (IFI), proposed and promoted by the World Road Association (PIARC), to address the non-comparability of friction data obtained using different devices, methods, and testing speeds. By employing an index system based on standardized parameters, heterogeneous measurements can be unified on a common basis [50].

#### 3.7.1. IFI Calculation Model and Method

According to the IFI calculation procedure recommended by PIARC, the texture parameter S_p_ can be derived from the macrotexture depth index, which is expressed as

(6) $$S_{p}=a+b\cdot \mathrm{MTD}$$

where S_p_ is the texture parameter; MTD is the mean texture depth; and a and bare empirical coefficients associated with the specific texture measurement method.

After obtaining the texture parameter, the pendulum-measured friction coefficient can be corrected for speed to calculate the friction index at the standard reference speed of 60 km/h. The expression is given as

(7) $$F_{60}=F\left(v\right)\cdot \exp\left[\frac{v-60}{S_{p}}\right]$$

where F_60_ is the friction index at the standard reference speed of 60 km/h; F(v)is the friction coefficient measured at the test speed v (km/h); and S_p_ is the texture parameter [51].

#### 3.7.2. IFI Result Analysis

Based on the previously obtained macrotexture depth (MTD) and water-film friction coefficient results, the IFI value was calculated using the computational model recommended by PIARC. During the pendulum friction tester process, the sliding speed of the rubber slider is set to 10 km/h. The measured value is substituted into the formula as F_10_ to calculate F_60_, which is then normalized (using a maximum value of 1.000 as the baseline) to facilitate cross-comparisons between different gradations [50].

The calculated International Friction Index (IFI) values for different gradation schemes are shown in Table 17.

The IFI results indicate pronounced differences in comprehensive skid resistance among the investigated gradation schemes, with relative IFI values ranging from 0.112 to 1.000. Among them, Gradation No. 11 (EA-10-11) achieves the maximum relative IFI value of 1.000. This gradation is characterized by passing rates of 75% for the 4.75 mm sieve, 61% for the 2.36 mm sieve, 34% for the 0.6 mm sieve, and 29.5% for the 0.3 mm sieve. These results demonstrate that optimal comprehensive skid resistance can be obtained when a moderate macrotexture depth (0.66 mm) is combined with a relatively high water-film friction coefficient (65.5). In contrast, Gradation No. 4 (EA-10-4) exhibits the minimum relative IFI value of 0.112. Although this gradation presents the largest macrotexture depth (0.76 mm) and a relatively high water-film friction coefficient (76.0), its overall skid resistance remains poor. This finding indicates that a single high macrotexture depth alone cannot guarantee superior comprehensive performance, and that coordinated matching between texture parameters and friction characteristics is more critical.

Notably, the No. 11 gradation achieved the highest relative IFI value (1.000) despite having the smallest MTD (0.66 mm), while the No. 4 gradation exhibited the lowest IFI (0.112) despite possessing the largest MTD (0.76 mm). The key characteristic of the No. 11 gradation lies in its combination of pass rates through the 2.36 mm (61%), 0.6 mm (34%), and 0.3 mm (29.5%) sieve apertures. This gradation structure creates a well-connected micro-pore network within the coarse aggregate skeleton, regulated by 0.6 mm and 0.3 mm particles. Although this structure exhibits average performance in terms of macro-texture depth (MTD), it effectively breaks up and expels the water film at the tire-road interface under wet conditions. This creates microscopic drainage channels, ensuring a high wet friction coefficient and ultimately achieving optimal overall skid resistance (IFI value). In contrast, No. 4 gradation exhibits a higher 4.75 mm pass rate (70%) and greater MTD (0.76 mm), but its 0.6 mm (30%) and 0.3 mm (26.5%) pass rates are lower, indicating insufficient fine aggregate filling. This results in a rich macrotexture, yet the internal pores are predominantly closed or isolated, lacking interconnected micro-drainage pathways. Under wet conditions, water films readily accumulate between the tire and road surface, unable to be effectively drained. This diminishes friction performance, leading to the lowest overall IFI rating.

Further analysis of the influence of the 2.36 mm sieve passing rate on IFI shows that when the passing rate is 52% (Gradations No. 1, 9, and 18), relative IFI values range from 0.162 to 0.446. At 55% (Gradations No. 2, 10, and 17), IFI values range from 0.194 to 0.646. When the passing rate is 58% (Gradations No. 3, 12, and 14), IFI values range from 0.408 to 0.600, indicating relatively favorable overall performance. At a passing rate of 61% (Gradations No. 4, 11, 13, and 19), IFI values span a wide range from 0.112 to 1.000, showing the greatest variability and suggesting that IFI within this interval is highly sensitive to variations in other sieve parameters. When the passing rate increases to 64% (Gradations No. 5, 8, and 15), IFI values range from 0.136 to 0.515, while at 67% (Gradations No. 6, 7, 16, and 20), IFI values range from 0.322 to 0.826. These results indicate that a 2.36 mm sieve passing rate in the range of 58–61% is conducive to achieving higher IFI values, provided that it is coordinated with optimized configurations of other sieves.

Analysis of the influence of the 0.6 mm sieve passing rate shows that when the passing rate is 30% (Gradations No. 1, 4, 8, 12, and 16), IFI values range from 0.112 to 0.826. When the passing rate is 34% (Gradations No. 2, 6, 9, 11, 14, 17, and 19), IFI values range from 0.194 to 1.000, representing the best overall performance. When the passing rate is 38% (Gradations No. 3, 5, 7, 10, 13, 15, 18, and 20), IFI values range from 0.136 to 0.677. These results suggest that a 0.6 mm passing rate near 34% is more favorable for improving comprehensive skid resistance, which is generally consistent with the conclusions drawn from the analyses of dry and water-film friction coefficients.

Correlation analysis between macrotexture depth and IFI indicates that for gradations with MTD values between 0.66 and 0.76 mm, IFI values range widely from 0.112 to 1.000, demonstrating that no simple positive correlation exists between macrotexture depth and IFI. Specifically, Gradation No. 11 achieves the highest IFI despite having the smallest MTD (0.66 mm), whereas Gradation No. 4 exhibits the largest MTD (0.76 mm) but the lowest IFI. These further highlights the importance of synergistic optimization between the texture parameter S_p_ and friction characteristics. The relationship between the water-film friction coefficient and IFI is similarly complex. For example, Gradation No. 12 shows the highest friction coefficient (81.5) but only a moderate IFI value of 0.570, whereas Gradation No. 5 exhibits the lowest friction coefficient (59.0) and also a relatively low IFI value (0.136). These observations confirm that extreme values of a single performance indicator cannot ensure optimal comprehensive skid resistance.

Overall, the variation trends of IFI are generally consistent with the results obtained from analyses of macrotexture depth, water-film friction coefficient, and dry friction coefficient. Gradation schemes with relatively large macrotexture depth and high water-film friction coefficient tend to exhibit higher texture parameters and friction indices, indicating that such gradations are capable of maintaining more stable skid resistance under wet and slippery conditions.

Based on the systematic analysis of texture depth, dry friction coefficient, water-film friction coefficient, and the International Friction Index (IFI) presented earlier, this study proposes a synergistic control mechanism involving a “macroscopic framework-microscopic pores” interaction that governs the skid resistance of epoxy–asphalt mixtures. This mechanism divides the internal structure of the mixture into two functional levels: a macroscopic load-bearing framework and a microscopic drainage network.

The macroscopic load-bearing framework is dominated by the sieve pass rates of 4.75 mm and 2.36 mm. The 4.75 mm sieve pass rate determines whether the coarse aggregate can form a stable, interlocking skeletal structure, and serves as the foundation for the formation of texture depth. A 2.36 mm sieve size serves as the boundary between coarse and fine aggregates; the pass rate at this size directly affects the degree of exposure of the aggregate edges and the density of interlocking, thereby playing a decisive role in the dry friction coefficient.

The micro-drainage network is dominated by the passing rates of 0.6 mm and 0.3 mm sieve sizes. Within the confined space of the macroscopic framework, the proportions of 0.6 mm and 0.3 mm particles determine the size, distribution, and connectivity of the microporosity within the framework’s interstices. The passing rate of the 0.6 mm sieve mesh acts as a connecting function, providing a rapid drainage pathway for the water film. The 0.3 mm sieve pass rate serves to stabilize the pore walls, preventing excessive filling with fine particles that could lead to pore blockage. The interconnected microporous network formed by the synergistic interaction of these two factors is critical for enhancing the water film friction coefficient and IFI.

These two structural levels do not exist in isolation. The macroscopic framework provides stable physical support and a spatial structure for the microscopic drainage network, while the microscopic drainage network enables the macroscopic framework to realize its anti-slip potential under wet and slippery conditions. This concept of coordinated optimization is universally applicable and can serve as a reference and methodological guide for the design of skid-resistant gradations in other types of asphalt mixtures.

In summary, as a comprehensive skid resistance indicator, IFI effectively identifies gradation deficiencies that cannot be captured by individual performance indices and provides a quantitative evaluation tool for multi-objective optimization of steel bridge deck pavements in hot and humid regions. Based on IFI analysis, gradation ranges of 58–61% for the 2.36 mm sieve, approximately 34% for the 0.6 mm sieve, 70–75% for the 4.75 mm sieve, and 26.5–29.5% for the 0.3 mm sieve can be considered priority ranges for optimizing comprehensive skid resistance. These findings corroborate the results of the integrated three-indicator analysis and further validate the rationality of the hierarchical control and synergistic optimization strategy.

## 4. Conclusions

Against the background of skid resistance requirements for steel bridge deck pavements in hot, humid, and rainy regions, this study systematically investigated the effects of different sieve passing rates on macrotexture depth, dry friction coefficient, and water-film friction coefficient of an EA-10 epoxy–asphalt mixture using an orthogonal experimental design. The International Friction Index (IFI) was further introduced to evaluate comprehensive skid resistance. On this basis, the functional roles of key sieves were clarified, and a gradation optimization method for skid-resistance-oriented epoxy–asphalt mixtures was proposed. The main conclusions are as follows:

1. Macrotexture depth is primarily governed by the coarse aggregate skeleton structure, with the 4.75 mm sieve passing rate acting as the dominant controlling factor and the 0.3 mm sieve passing rate playing an important regulating role. When the 4.75 mm passing rate is approximately 70% and the 0.3 mm passing rate is around 26.5%, a stable interlocking skeleton of coarse aggregates can be formed, which is favorable for the development of pavement macrotexture.
2. The dry and water-film friction coefficients exhibit consistent response patterns to gradation structure and are mainly controlled by the 2.36 mm and 0.6 mm sieve passing rates. Among these, 2.36 mm has a significant effect on the dry friction coefficient at the critical significance level. The 2.36 mm passing rate determines the morphology of the surface skeleton and the degree of aggregate angularity exposure, while the 0.6 mm passing rate regulates the skeleton void structure and plays a key role in friction performance under both dry and wet conditions.
3. Based on the integrated analysis of the three skid resistance indicators, a coordinated control scheme for key sieves in skid-resistance-oriented epoxy–asphalt mixtures is proposed. When the 2.36 mm passing rate is controlled within 58–61% and the 0.6 mm passing rate is maintained at approximately 34%, in combination with a 4.75 mm passing rate of about 70% and a 0.3 mm passing rate of about 26.5%, the mixture can achieve superior comprehensive skid resistance.
4. IFI evaluation results demonstrate that synergistic matching between macrotexture depth and friction performance is decisive for comprehensive skid resistance, and extreme values of a single skid resistance indicator cannot fully represent the overall skid resistance level of the mixture. The graded aggregate exhibits more stable overall skid resistance through the synergistic interaction between a stable macro-scale framework and interconnected micro-scale voids formed by key fine aggregate sieve apertures such as 0.6 mm and 0.3 mm.

## Figures and Tables

**Table 1:** Technical specifications for 70# base asphalt.

Testing Items	Unit	Technical Requirement	Measured Value	Experiment Methods
Penetration (25 °C, 5 s, 100 g)	0.1 mm	60~80	62	T0604-2011
Softening point (TR&B)	°C	≥47	48.0	T0606-2011
Ductility (10 °C, 5 cm/min)	cm	≥20	35	T0605-2011
Ductility (15 °C, 5 cm/min)	cm	≥100	>100	T0605-2011
Flash point	°C	≥260	336	T0611-2011
Density (15 °C)	g/cm^3^	≥1.000	1.028	T0603-2011
Residue after the Rolling Thin Film Oven Test (RTFOT) (163 °C, 85 min)				
Mass change	%	8.367	8	T0610-2011
Residual ductility (10 °C, 5 cm/min)	cm	9.41	9	T0605-2011
Residual penetration ratio (25 °C)	%	10.45	10	T0604-2011

**Table 2:** Physical properties and technical specifications of the epoxy resin.

Testing Items	Unit	Technical Requirement	Test Results	Experiment Methods
Viscosity (23 °C)	/	1000~5000	2215	GB/T12007.4-1989 [35]
Specific gravity (23 °C)	g/cm^3^	1.00~1.20	1.138	T0603-1993
Epoxy equivalent	/	190~220	200	GB/T4612-1984 [36]
Flash point	°C	>220	235	T0611-1993
Appearance	/	Pale yellow transparent liquid	Pale yellow transparent liquid	Visual inspection

**Table 3:** Physical properties and technical specifications of the curing agent.

Testing Items	Unit	Technical Requirement	Test Results	Experiment Methods
Amine value	mg, KOH/g	150~200	160	T0606-2000
Viscosity (23 °C)	/	100~800	191	GB/T12007.4-1989
Specific gravity (23 °C)	g/cm^3^	0.80~1.00	0.848	T0603-1993
Flash point	°C	>145	169	T0611-1993
Appearance	/	Light yellowish-brown liquid	Light yellowish-brown liquid	Visual inspection

**Table 4:** Technical specifications for coarse aggregate.

Testing Items	Technical Requirement	Test Results	Experiment Methods
Los Angeles abrasion rate, %	≤16	11.2	JTG E42-2005(T 0317-2005)
Polish value, PSV	≥44	50	JTG E42-2005 (T0321-2005)
Needle and flake content, %	≤5	3.8	JTGE42-2005 (T0312-2005)
Crush value, %	≤12	9.1	JTG E42-2005 (T0316-2005)
Adhesion to asphalt, Grade	≥4	5	JTJ E20-2011 (T0616-1993)
Water absorption rate, %	≤1.5	0.61	JTG E42-2005 (T0308-2005)
Apparent density, g·cm^−3^	≥2.70	2.946	JTG E42-2005 (T0308-2005)
Sturdiness, %	≤5	1	JTG E42-2005 (T0314-2000)
Soft rock content, %	≤1	0.1	JTG E42-2005 (T0320-2000)
Particle content <0.075, %	≤0.8	0.1	JTG E42-2005 (T0303-2005)

**Table 5:** Technical specifications for fine aggregates.

Testing Items	Unit	Technical Requirement	Test Results	Experiment Methods
Apparent relative density	g·cm^−3^	≥2.70	2.926	JTG E42-2005 T0328
Sturdiness	%	≤12	2	JTG E42-2005 T0340
Sand equivalent	%	≥70	88	JTG E42-2005 T0334
Methylene blue value	g/kg	≤2.5	0.8	JTG E42-2005 T0349
Angularity (flow time)	s	≥30	30.3	JTG E42-2005 T0345

**Table 6:** Technical specifications for mineral powder.

Testing Items	Technical Requirement	Test Results	Experiment Methods
Apparent density, g·cm^−3^	≥2.50	2.764	JTG E42-2005 T0352
Appearance	No clumping	No clumping	——
Moisture content, %	≤1	0.2	JTG 051-1993 T0103
Hydrophilicity coefficient	≤1	0.81	JTG E42-2005 T0353
Plasticity Index, %	≤4	3.8	JTG E42-2005 T0355
Stability	No deteriorating	No change	JTG E42-2005 T0355

**Table 7:** Orthogonal test factor and level gradation table.

Number	Sieve Aperture Size/mm (Passing Rates/%)							
	9.5	4.75	2.36	1.18	0.6	0.3	0.15	0.075
EA-10-1	100	70	52	42	30	23.5	15.5	8.5
EA-10-2	100	70	55	47	34	29.5	21.5	8.5
EA-10-3	100	70	58	42	38	29.5	18.5	10.5
EA-10-4	100	70	61	52	30	26.5	21.5	10.5
EA-10-5	100	70	64	47	38	26.5	15.5	12.5
EA-10-6	100	70	67	52	34	23.5	18.5	12.5
EA-10-7	100	75	67	42	38	26.5	21.5	8.5
EA-10-8	100	75	64	52	30	29.5	18.5	8.5
EA-10-9	100	75	52	47	34	26.5	18.5	10.5
EA-10-10	100	75	55	52	38	23.5	15.5	10.5
EA-10-11	100	75	61	42	34	29.5	15.5	12.5
EA-10-12	100	75	58	47	30	23.5	21.5	12.5
EA-10-13	100	80	61	47	38	23.5	18.5	8.5
EA-10-14	100	80	58	52	34	26.5	15.5	8.5
EA-10-15	100	80	64	42	34	23.5	21.5	10.5
EA-10-16	100	80	67	47	30	29.5	15.5	10.5
EA-10-17	100	80	55	42	30	26.5	18.5	12.5
EA-10-18	100	80	52	52	38	29.5	21.5	12.5
EA-10-19	100	75	61	47	34	26.5	18.5	10.5
EA-10-20	100	80	67	52	38	29.5	21.5	12.5

**Table 8:** Textural depth of epoxy–asphalt mixture.

**Gradation Number**	1	2	3	4	5	6	7	8	9	10
**Texture Depth (mm)**	0.73	0.72	0.70	0.76	0.75	0.69	0.71	0.69	0.70	0.68
**Gradation Number**	11	12	13	14	15	16	17	18	19	20
**Texture Depth (mm)**	0.66	0.69	0.70	0.69	0.69	0.67	0.68	0.69	0.71	0.67

**Table 9:** Analysis of range results for macrotexture depth.

Indicator	Factor A	Factor B	Factor C	Factor D	Factor E	Factor F	Factor G
K_1_	4.35	2.12	4.17	4.22	4.18	4.18	4.24
K_2_	4.84	2.08	4.94	4.86	5.00	4.87	4.91
K_3_	4.79	2.08	4.87	4.90	4.80	4.93	4.83
K_4_		2.83					
K_5_		2.13					
K_6_		2.74					
k_1_	0.725	0.707	0.695	0.703	0.697	0.697	0.707
k_2_	0.691	0.693	0.706	0.694	0.714	0.696	0.701
k_3_	0.684	0.693	0.696	0.700	0.686	0.704	0.690
k_4_		0.708					
k_5_		0.710					
k_6_		0.685					
Range	0.041	0.025	0.011	0.009	0.029	0.009	0.017

**Table 10:** Variance analysis table for macrotexture depth.

Sources of Variance	Sum of Squares	Degrees of Freedom	Mean Square	F-Value	Significance
A	0.006	2	0.003	111.575	0.009
B	0.001	5	0.000	10.195	0.092
C	0.000	2	0.000	7.163	0.123
D	0.001	2	0.000	11.382	0.081
E	0.003	2	0.001	50.209	0.020
F	0.001	2	0.000	17.801	0.053
G	0.001	2	0.000	11.165	0.082
Error	5.143 × 10^−5^	2	2.571 × 10^−5^		

**Table 11:** Dry friction coefficient of epoxy–asphalt mixture.

**Gradation Number**	1	2	3	4	5	6	7	8	9	10
**Dry Friction Coefficient**	80	69	69	79.5	65	69.5	65	70.5	72.5	69.5
**Gradation Number**	11	12	13	14	15	16	17	18	19	20
**Dry Friction Coefficient**	67.5	85	70	81.5	65	70.5	79	68.5	70.5	66

**Table 12:** Analysis of range results for dry friction coefficient.

Indicator	Factor A	Factor B	Factor C	Factor D	Factor E	Factor F	Factor G
K_1_	432.0	221.0	425.5	464.5	439.0	434.0	436.0
K_2_	500.5	217.5	502.5	495.5	513.0	501.0	496.5
K_3_	500.5	235.5	505.0	473.0	481.0	498.0	500.5
K_4_		287.5					
K_5_		200.5					
K_6_		271.0					
k_1_	72.0	73.7	70.9	77.4	73.2	72.3	72.7
k_2_	71.5	72.5	71.8	70.8	73.3	71.6	70.9
k_3_	71.5	78.5	72.1	67.6	68.7	71.1	71.5
k_4_		71.9					
k_5_		66.8					
k_6_		67.8					
Range	0.5	11.7	1.2	9.9	4.6	1.2	1.7

**Table 13:** Variance analysis table for dry friction coefficient.

Sources of Variance	Sum of Squares	Degrees of Freedom	Mean Square	F-Value	Significance
A	3.324	2	1.662	0.824	0.548
B	253.116	5	50.623	25.102	0.039
C	19.521	2	9.761	4.840	0.171
D	287.608	2	143.804	71.308	0.014
E	71.260	2	35.630	17.668	0.054
F	1.315	2	0.657	0.326	0.754
G	11.964	2	5.982	2.966	0.252
Error	4.033	2	2.017		

**Table 14:** Water-film friction coefficient of epoxy–asphalt mixture.

**Gradation Number**	1	2	3	4	5	6	7	8	9	10
**Water-Film Friction Coefficient**	75.5	62	65.5	76	59	63	59.5	68	68.5	64
**Gradation Number**	11	12	13	14	15	16	17	18	19	20
**Water-Film Friction Coefficient**	65.5	81.5	64.5	76	59.5	65	73	64.5	67	60.5

**Table 15:** Analysis of range results for water-film friction coefficient.

Indicator	Factor A	Factor B	Factor C	Factor D	Factor E	Factor F	Factor G
K_1_	401.0	208.5	398.5	439.0	408.0	405.0	405.5
K_2_	474.0	199.0	467.5	461.5	479.0	469.5	465.5
K_3_	463.0	223.0	472.0	437.5	451.0	463.5	467.0
K_4_		273.0					
K_5_		186.5					
K_6_		248.0					
k_1_	66.8	69.5	66.4	73.2	68.0	67.5	67.6
k_2_	67.7	66.3	66.8	65.9	68.4	67.1	66.5
k_3_	66.1	74.3	67.4	62.5	64.4	66.2	66.7
k_4_		68.3					
k_5_		62.2					
k_6_		62.0					
Range	1.6	12.3	1.0	10.7	4.0	1.3	1.1

**Table 16:** Variance analysis table for water-film friction coefficient.

Sources of Variance	Sum of Squares	Degrees of Freedom	Mean Square	F-Value	Significance
A	3.064	2	1.523	0.675	0.597
B	316.853	5	63.371	28.076	0.035
C	21.912	2	10.956	4.854	0.171
D	340.184	2	170.092	75.357	0.013
E	39.849	2	19.924	8.827	0.102
F	0.661	2	0.331	0.146	0.872
G	8.830	2	4.415	1.956	0.338
Error	4.514	2	2.257		

**Table 17:** IFI Calculation Results for Different Gradation Schemes.

Gradation Number	MTD/mm	Water-Film Friction Coefficient	Texture Parameter S_p_	Relative IFI
1	0.73	75.5	1.54	0.162
2	0.72	62	1.53	0.194
3	0.7	65.5	1.5	0.408
4	0.76	76	1.56	0.112
5	0.75	59	1.55	0.136
6	0.69	63	1.49	0.54
7	0.71	59.5	1.51	0.322
8	0.69	68	1.49	0.515
9	0.7	68.5	1.5	0.446
10	0.68	64	1.48	0.646
11	0.66	65.5	1.46	1.0
12	0.69	81.5	1.49	0.57
13	0.7	64.5	1.5	0.442
14	0.69	76	1.49	0.6
15	0.69	59.5	1.49	0.45
16	0.67	65	1.47	0.826
17	0.68	73	1.48	0.506
18	0.69	64.5	1.49	0.442
19	0.71	67	1.51	0.297
20	0.67	60.5	1.47	0.677

## Data Availability

The original findings presented in this study are included in this article. If you have any further questions, please contact the corresponding author.
